# Familial Linkage and Association of the *NR3C1* Gene with Type 2 Diabetes and Depression Comorbidity

**DOI:** 10.3390/ijms231911951

**Published:** 2022-10-08

**Authors:** Mutaz Amin, Shumail Syed, Rongling Wu, Teodor Tudorel Postolache, Claudia Gragnoli

**Affiliations:** 1 Institut National de la Santé et de la Recherche Médicale (INSERM), US14-Orphanet, 75014 Paris, France; 2 Department of Biochemistry and Molecular Biology, Faculty of Medicine, Al-Neelain University, Khartoum 11121, Sudan; 3 Division of Endocrinology, Department of Medicine, Creighton University School of Medicine, Omaha, NE 68124, USA; 4 Department of Public Health Sciences, Penn State College of Medicine, Hershey, PA 17033, USA; 5 Department of Statistics, Penn State College of Medicine, Hershey, PA 17033, USA; 6 Mood and Anxiety Program, Department of Psychiatry, University of Maryland School of Medicine, Baltimore, MD 21201, USA; 7 Rocky Mountain Mental Illness Research Education and Clinical Center (MIRECC), Veterans Integrated Service Network (VISN) 19, Military and Veteran Microbiome: Consortium for Research and Education (MVM-CoRE), Denver, CO 80246, USA; 8 Mental Illness Research Education and Clinical Center (MIRECC), Veterans Integrated Service Network (VISN) 5, VA Capitol Health Care Network, Baltimore, MD 21090, USA; 9 Molecular Biology Laboratory, Bios Biotech Multi-Diagnostic Health Center, 00197 Rome, Italy

**Keywords:** nuclear receptor subfamily 3 group C member, *NR3C1*, glucocorticoid receptor, GR, major depressive disorder, MDD, type 2 diabetes, T2D, cortisol, hypothalamic-pituitary-adrenal axis, HPA axis, comorbid, comorbidity, mental-metabolic

## Abstract

Impairment in the hypothalamic-pituitary-adrenal (HPA) axis and cortisol pathway may be major contributing factors to the common pathogenesis of major depressive disorders (MDD) and type 2 diabetes (T2D). A significant player in the neuroendocrine HPA axis and cortisol response is the glucocorticoid receptor (GR), which is encoded by the nuclear receptor subfamily 3 group C member (*NR3C1*) gene. Variants in the *NR3C1* gene have been reported in patients with MDD and obesity and found to confer reduced risk for quantitative metabolic traits and T2D in Cushing syndrome; variants have not been reported in T2D and MDD-T2D comorbid patients. We studied 212 original Italian families with a rich family history for T2D and tested 24 single nucleotide polymorphisms (SNPs) in the *NR3C1* gene for linkage to and linkage disequilibrium (LD) with T2D and MDD across different inheritance models. We identified a total of 6 novel SNPs significantly linked/in LD to/with T2D (rs6196, rs10482633, rs13186836, rs13184611, rs10482681 and rs258751) and 1 SNP (rs10482668) significantly linked to/in LD with both T2D and MDD. These findings expand understanding of the role that *NR3C1* variants play in modulating the risk of T2D-MDD comorbidity. Replication and functional studies are needed to confirm these findings.

## 1. Introduction

Major depressive disorder (MDD) and type 2 diabetes (T2D) are two common complex multifactorial disorders that share several genetic and environmental risk factors such as hypercortisolism and related genes’ risk variants within the stress response and neuroendocrine hypothalamic-pituitary-adrenal axis (HPA) [1]. Stress hyperactivates the HPA axis by triggering the secretion of the hypothalamic corticotropin-releasing hormone (CRH). CRH stimulates the anterior pituitary to release the adrenocorticotropin hormone (ACTH), which causes the adrenal secretion of cortisol [2]. Depression is associated with increased cortisol levels [3], and hypercortisolism contributes to serotonin dysfunction and mood disturbances [4] as well as to hyperglycemia and insulin resistance [5,6,7], which are precursors of T2D [8]. Impairment in the cortisol pathway may be a major contributing factor to the common pathogenesis of MDD and T2D [9]. A significant player in the cortisol pathway is the glucocorticoid receptor (GR), which is expressed ubiquitously and mediates the HPA axis negative feedback at the hypothalamus and pituitary level [10], and which is encoded by the *NR3C1* gene [11]. Epigenetic changes of the *NR3C1* gene affect coping style and depression vulnerability [12,13,14]. Variants in the *NR3C1* gene have been reported to contribute to: the risk for MDD [15]; antidepressants response [16]; metabolic traits or phenotypes, such as insulin resistance [17]; reduced risk for T2D in Cushing syndrome [18]; reduced risk for quantitative metabolic traits (e.g., fasting plasma glucose, glycated hemoglobin), in subjects stratified for the metabolic ineffective cortisone, a cortisol metabolite [19]; and increased risk for T2D in adults with decreased birth length [20]. Of note, activation of the glucocorticoid receptor in mice by 17-hydroxyprogestrone and dexamethasone mediates hyperglycemia/insulin resistance [21] and depression-like state [22] respectively. To our knowledge, no prior study has reported a positive risk role for the qualitative familial T2D and MDD phenotypes. We thus hypothesized that the *NR3C1* gene might underlie the MDD-T2D comorbidity and aimed at investigating whether *NR3C1* variants might predispose to familial T2D, MDD, and/or MDD-T2D comorbidity in affected families.

## 2. Results

**Figure 1:**
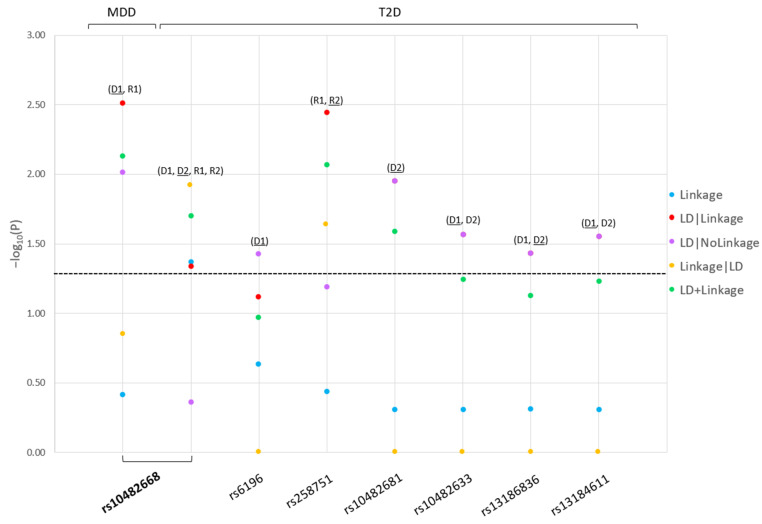
Major depressive disorder and type 2 diabetes *NR3C1* risk single nucleotide polymorphisms linkage and linkage disequilibrium analysis results. **Legend:** For each significant risk single nucleotide polymorphism (SNP) in the *NR3C1* gene, we present the –log10(P) as a function of each test statistic (Linkage, Linkage Disequilibrium (LD)|Linkage, LD|NoLinkage, Linkage|LD, and LD + Linkage) and label the inheritance model: D1: dominant, complete penetrance, D2: dominant, incomplete penetrance, R1: recessive, complete penetrance, R2: recessive, incomplete penetrance. For each single nucleotide polymorphism (SNP), we present the most significant test statistics (underlined) across the significant models. The bolded SNP is comorbid for major depressive disorder and type 2 diabetes.

We identified in our cohort of 212 Italian families novel linkage, linkage disequilibrium (LD, i.e., linkage + association), and association of *NR3C1*-variants to both T2D and T2D-MDD comorbidity. Of 24 studied variants in the *NR3C1* gene, 6 independent single nucleotide polymorphisms (SNPs) were significantly linked to/in LD or associated with T2D (rs6196, rs10482633, rs13186836, rs13184611, rs10482681, and rs258751), and 1 SNP (rs10482668) significantly linked to/in LD or associated with both T2D and MDD (*p* < 0.05). (Figure 1). The SNPs were statistically significant across different models: for T2D: D1 (rs6196, rs10482681, rs10482668, rs13186836, and rs13184611), D2 (rs10482681, rs10482668, rs10482633, rs13186836, and rs13184611), R1 (rs258751 and rs10482668), R2 (rs258751 and rs10482668); for MDD: D1 and R1 (rs10482668); and the latter rs10482669 resulted comorbid for MDD-T2D (Table 1).

## 3. Discussion

Cortisol is a pleiotropic glucocorticoid that upon binding to its widely distributed GR, mediates the adaptive, physiologic, or pathologic responses to chronic stress, such as mood changes and hyperglycemia [24,25,26,27]. Genes encoding components throughout the HPA can therefore be considered candidate genes for mood disorders (e.g., depression) and metabolic abnormalities (e.g., type 2 diabetes). We have previously reported that the *CRHR2* [28] and the melanocortin receptor genes (*MC1R*-*MC5R*) [29] are linked to and associated with the comorbidity of T2D and MDD. In this study, we extended this linkage and association to the glucocorticoid receptor gene (*NR3C1*), which is an important component of the cortisol pathway. We have reported finding six *NR3C1* variants that are significantly linked/in LD to/with T2D and one variant significantly linked/in LD to/with both MDD and T2D across different inheritance models. Except for rs6196, these variants are novel, and have not previously been reported with either MDD and/or T2D. Of note, the risk allele (A) of the T2D-risk rs6196 variant in our study was previously reported in a haplotype in Caucasian patients with MDD [23], but this association was not consistent in other studies from both similar [30,31,32] and different ethnicities [33], probably due to different sampled sizes. Of note, heterozygous genotypes including the same T2D-risk variant (rs6196, allele A) were previously found to be associated with metabolic syndrome [34]. Moreover, in low-birth weight children, those with a haplotype carrying the risk allele A and 1 cm of decreased length at birth had higher predisposition for impaired glucose tolerance or T2D later in life [20]. As glucose intolerance and metabolic syndrome are, respectively, a pre-diabetes and T2D associated phenotypes, the above-mentioned findings are consistent with the results from our study. Of interest, a study reported that the *NR3C1* variant was associated with reduced risk of T2D in patients with Cushing syndrome [18].

The mechanism by which these variants modulate the risk of T2D and/or MDD could not be fully determined. In fact, our in silico analysis (splicing [SpliceAI] [35], transcription-factor binding [SNPnexus] [36], SNP function prediction [37], regulatory potential [RegulomeDB] [38], and miRNA binding [mirSNP] [39]) predicted no functional impact on the GR protein. However, upon subsequent analysis, in the Italian Tuscany population, the two T2D-risk variants rs6196 and rs10482633 were in significant LD with the *NR3C1*-variant rs41423247 (D’ = 0.9, *p* < 0.0001 and D’ = 1, *p* < 0.0001, respectively), which is known to be associated with MDD [40], pregnancy-related hyperglycemia [41], metabolic syndrome [34,42], and obesity and impaired glucose tolerance [34], thereby highlighting the potential role of *NR3C1* and its reported genetic variants in the mental-metabolic comorbidity. If the variants in our study are found to be associated with structural or functional impairment of NR3C1 and defective binding to circulating cortisol, it could potentially explain the MDD-related [4] and T2D-related [5,6,7] traits associated with hypercortisolism. One plausible explanation might be the loss of negative feedback inhibition mediated by NR3C1 at the level of the hypothalamus and pituitary gland. This might result in chronic hyperactive hypercortisolism and the consequential damaging effects that it may cause. It also is possible that the risk role played by the genetic variations detected in our study is indirectly mediated by the presence of LD with other nearby variants; however, functional studies are needed to fully elucidate such mechanisms.

## 4. Materials and Methods

We recruited 212 families originating from the Italian peninsula ascertained for T2D and rich family history of T2D. The families were also afflicted with several cases of MDD, diagnosed according to DSM-IV criteria. Given that the families are three-generation Italians, that uncertain paternity cases as well as adopted cases and identical twins were excluded, and especially that the families derive from the gene pool of a peninsular population, the chance of identifying genetic markers in linkage and LD (i.e., linkage + association) for the ascertained phenotypes is high. We genotyped 24 SNPs within the *NR3C1* gene using microarray. We first excluded genotyping and Mendelian errors using PLINK [43]. We then analyzed the SNPs for 2-point parametric testing (for T2D and MDD) using Pseudomarker [44], which is a powerful tool for joint exploration of linkage and LD. Two-point analysis entails testing each SNP individually against the putative disease locus. We tested the SNPs for parametric linkage to and/or LD with T2D and MDD across the following models: dominant with complete penetrance (D1), dominant with incomplete penetrance (D2), recessive with complete penetrance (R1), and recessive with incomplete penetrance (R2), *p*-values <0.05 were considered significant. To evaluate the presence of LD blocks (correlation of r2 ≥ 0.9) among the risk variants, we analyzed the LD matrix of the Tuscany Italian population derived from the 1000 Genomes Project (https://www.internationalgenome.org/data-portal/population/TSI (accessed on 28 September 2021)) [45]. This analysis allows the identification of potential risk variants within the same LD block and provides information regarding the gene components conferring linkage or LD for each disorder or their comorbidity. If there are no LD blocks present among the risk variants, the variants will be defined as “independent”, thus contributing to the phenotype under study independently from each other as well as being potentially subject to recombination reciprocally from each other.

## 5. Conclusions

We report novel linkage, linkage + association, and association of the *NR3C1* gene to T2D and T2D-MDD comorbidity. Our results expand the understanding of the role that *NR3C1* variants play in modulating the risk of T2D-MDD comorbidity. Replication and functional studies are needed to confirm these findings.

## Figures and Tables

**Table 1:** Risk single nucleotide polymorphisms in *NR3C1* gene linked/in linkage disequilibrium to/with major depressive disorder and/or type 2 diabetes.

Disease	Model ^1^	SNP	Position	Ref	Alt	Risk Allele	Consequence	LD Block	Reported in MDD or T2D?
**MDD**	D1, R1	**rs10482668**	143313762	T	A	T	Intronic	Independent	Novel
**T2D**	D1	rs6196	143281925	A	G	A	Synonymous	Independent	Yes (MDD) [23]
	R1, R2	rs258751	143282715	G	A	G	Synonymous	Independent	Novel
	D2	rs10482681	143299858	A	C	C	Intronic	Independent	Novel
	D1, D2, R1, R2	**rs10482668**	143313762	T	A	T	Intronic	Independent	Novel
	D1, D2	rs10482633	143370968	T	G	G	Intronic	Independent	Novel
	D1, D2	rs13186836	143418420	T	C	C	Intronic	NA	Novel
	D1, D2	rs13184611	143422369	C	T	T	Intronic	NA	Novel

**Legend:**^1^ Models: D1: dominant, complete penetrance, D2: dominant, incomplete penetrance, R1: recessive, complete penetrance, R2: recessive, incomplete penetrance. The comorbid SNP is highlighted in bold. **Abbreviations:** SNP, single nucleotide polymorphism; LD, linkage disequilibrium; MDD, major depressive disorder, T2D, type 2 diabetes.

## Data Availability

The study data are available on reasonable request, and due to lacking specific patients’ consent and privacy restrictions, they are not publicly available.
